# Comparison of investigator-delineated gross tumour volumes and quality assurance in pancreatic cancer: Analysis of the on-trial cases for the SCALOP trial

**DOI:** 10.1016/j.radonc.2016.07.002

**Published:** 2016-08

**Authors:** Emmanouil Fokas, Emiliano Spezi, Neel Patel, Chris Hurt, Lisette Nixon, Kwun-Ye Chu, John Staffurth, Ross Abrams, Somnath Mukherjee

**Affiliations:** a Department of Oncology, CRUK/MRC Institute for Radiation Oncology, University of Oxford, UK; b School of Engineering, Cardiff University, UK; c Oxford University Hospital NHS Foundation Trust, UK; d Wales Cancer Trials Unit, Centre for Trials Research, Cardiff University, UK; e Institute of Cancer and Genetics, Cardiff University, UK; f Cardiff NCRI RTTQA Centre, Velindre NHS Trust, UK; g Department of Radiation Oncology, Rush University Medical Center, Chicago, USA

**Keywords:** Radiotherapy, Pancreas, Prospective trial, Conformity index, Quality assurance

## Abstract

**Background and purpose:**

We performed a retrospective central review of tumour outlines in patients undergoing radiotherapy in the SCALOP trial.

**Materials and methods:**

The planning CT scans were reviewed retrospectively by a central review team, and the accuracy of investigators’ GTV (iGTV) and PTV (iPTV) was compared to the trials team-defined gold standard (gsGTV and gsPTV) using the Jaccard Conformity Index (JCI) and Geographical Miss Index (GMI). The prognostic value of JCI and GMI was also assessed. The RT plans were also reviewed against protocol-defined constraints.

**Results:**

60 patients with diagnostic-quality planning scans were included. The median whole volume JCI for GTV was 0.64 (IQR: 0.43–0.82), and the median GMI was 0.11 (IQR: 0.05–0.22). For PTVs, the median JCI and GMI were 0.80 (IQR: 0.71–0.88) and 0.04 (IQR: 0.02–0.12) respectively. Tumour was completely missed in 1 patient, and ⩾ 50% of the tumour was missed in 3. Patients with JCI for GTV ⩾ 0.7 had 7.12 (95% CIs: 1.83–27.67, *p* = 0.005) higher odds of progressing by 9 months in multivariate analysis. Major deviations in RT planning were noted in 4.5% of cases.

**Conclusions:**

Radiotherapy workshops and real-time central review of contours are required in RT trials of pancreatic cancer.

In modern radiotherapy trials, a high quality Radiotherapy (RT) Trial Quality Assurance programme (RTQA) is essential to facilitate administration of high quality RT [1], [2]. Previous studies have reported an adverse impact of violation in RT protocol on the overall survival of patients [1], [2], [3].

A RTQA protocol should incorporate detailed contouring instructions, outlining atlases and benchmark cases to provide training to radiation oncologists and confirm their ability to execute treatment according to the protocol guidelines [4]. However, the previous RTQA studies have largely reported pre-trial benchmark case outlining and plans, and on-trial reports have focused on the quality of RT plans rather than the quality of tumour delineation [4]. Quality of tumour delineation is likely to be particularly important in pancreatic cancer as tumours have ill-defined margins and remain closely related to critical organs at risk (OARs), exposing patients both to risk of geographic miss as well as normal tissue toxicity [5].

The Selective Chemoradiotherapy (CRT) in Advanced Localized pancreatic cancer study (SCALOP, EudraCT No: 2008-001394-1) was a randomized phase II trial [6]. Patients were treated with induction chemotherapy with gemcitabine/capecitabine followed by randomization to either gemcitabine- or capecitabine-based CRT [5]. All patients provided written informed consent and the trial protocol was approved by the UK Medicines and Healthcare Products Regulatory Agency (MHRA) and a multi-centre research ethics committee. In total, 114 patients from 28 centres participated, and 74 patients received CRT. Radiation was given conformally to a dose of 50.4 Gy in 28 fractions; concomitant gemcitabine was given at a dose of 300 mg/m2 weekly, and capecitabine was 830 mg/m2 administered twice daily on days of RT. We have previously reported the main trial outcome [6], and more recently demonstrated considerable variation in GTV delineation among the participating investigators from pre-trial RTTQA programme [7].

In the present study, we have retrospectively reviewed the individual contours of patients entered in the trial using planning CT scans collected prospectively during the trial, and the RT plans were reviewed against protocol-defined constraints. The purpose of the present work was to examine: (1) the comparison of tumour volumes delineated by trial investigators with “gold standard” ones re-outlined by central team; (2) the utility of Jaccard Conformity Index (JCI) and Geographical Miss Index (GMI) in predicting the primary clinical endpoint of the SCALOP1 trial; (3) the compliance of the individual investigators to all aspects of dose prescription and RT protocol.

## Materials and methods

The planning CT scans of patients undergoing RT as part of SCALOP trial, including contoured tumour and OAR volumes, 3D dose cube and the Planning Assessment Form (PAF) were submitted by the investigators to the central RTQA office. The PAF (Supplementary Fig. 1) had been developed as a trial-specific, patient-specific form where the investigators were instructed to manually enter the dose to tumour and OAR in a given patient. The trial-specific dose constraints were pre-printed on the PAF as aid-memoire, to minimize the chances of protocol violation.

### Review of tumour outlines of the on-trial cases

The planning CT scans from all patients were reviewed by the central team that consisted of 2 radiation oncologists with experience in pancreatic cancer RT contouring (SM, EF), including the Chief Investigator (SM) and a radiologist with experience in pancreatic radiology (NP). From the total of 74 patients randomised within the trial, the intravenous (iv) contrasted planning CT in 62 patients were considered to be of sufficient diagnostic quality to allow re-outlining of the pancreatic tumour without the need for further diagnostic imaging. Two of these patients died prior to receiving any radiotherapy and so were excluded from these analyses. The tumours were outlined independently, without considering the outline of the original investigator. ‘Gold standard’ set of reference structures (gsGTV, gsPTV) were created for each patient following assessment of and consensus for each of the contoured on-trial cases (*n* = 60) by the central review team. The original investigator defined volumes (iGTV and iPTV) were then compared against gsGTV and gsPTV using geometric analysis [8].

### Geometric analysis of contours

For geometric analysis, DICOM-RT datasets from individual patients were uploaded into the Computational Environment for Radiotherapy Research (CERR) [9], a Matlab-based open source application capable of processing radiotherapy treatment planning data in DICOM^2^ format including volumes of interest and dose maps. Whole volume Jaccard Conformity Index (JCI) and Geographic Miss Index (GMI) [8] were generated for both GTV and PTV comparison as described before [10], [11]. JCI is defined as the volume of the gold standard contour encompassed by the investigator contour as a fraction of their combined volume. A JCI of 1 represents total agreement, and JCI of 0 represents no agreement. GMI is defined as the volume of gold standard contour missed by the investigator contour, as a fraction of the gold standard contour. A GMI of 0 represents no miss, whereas a GMI of 1 represents that all of the gold standard volume has been missed by the investigator. A good investigator contour will have a JCI close to 1 and a GMI close to 0. These indices have been defined further in Supplementary Table 1.

### Review of RT plans

The Visualization and Organization of Data for Cancer Analysis programme (VODCA 4; Medical Software Solutions, Hagendorn, Switzerland), a commercially available software package developed for the analysis of multicenter radiotherapy trials, was used to centrally review the DICOM-RT planning datasets for each submitted contour [7]. We investigated the plan quality and adherence to dose–volume constraints. We also examined whether PAF was completed correctly by the participating investigators by comparing the dose values documented in PAF with those generated from VODCA.

### Statistical analysis

Statistical analyses were performed with the Stata 14 SE package (StatCorp LP, College Station, Texas, USA) according to a pre-specified analysis plan. The Shapiro–Wilk test was used to assess normality of variables before presenting summary statistics and correlations. The sensitivity and specificity of the GMI and JCI in predicting the primary endpoint of the SCALOP1 trial (progression free survival at 9 months after registration into the trial) were assessed using ROC curves to find best cut offs and logistic regression was used to allow univariable and multivariable analysis and calculation of odds ratios. The multivariable model included all variables thought a priori to have prognostic value.

## Results

### Geometric analysis of contours

**Fig. 1:**
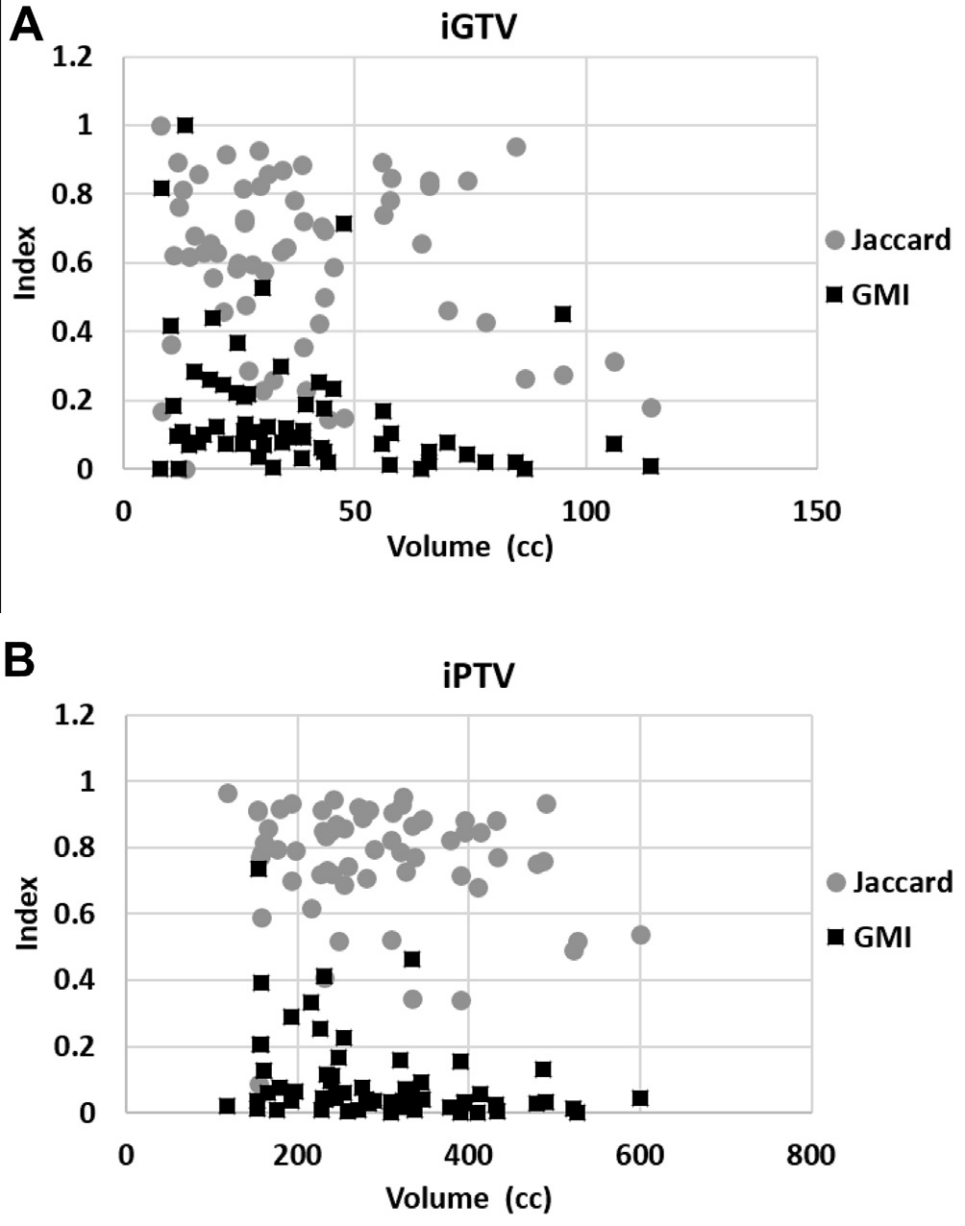
Scatter plot showing the correlation of the Geographical Miss Index (GMI) and the Jaccard Conformity Index (JCI) with (A) the investigator gross tumour volumes (iGTVs) and (B) the investigator planning target volumes (iPTVs).

The iGTV and iPTV volumes, with the corresponding GMI and JCI data for the *n* = 60 cases are shown in Fig. 1. The median volumes of gsGTV and gsPTV were 28.0 cm^3^ (IQR: 15.7–36.2) and 256.9 cm^3^ (IQR: 195.0–313.4), respectively. In comparison, the median iGTVs and iPTVs were 33.2 cm^3^ (IQR: 21.3–46.4) and 277.5 cm^3^ (IQR: 220.8–346.2), respectively. The median whole volume JCI for GTV was 0.64 (IQR: 0.43–0.82), and the median GMI was 0.11 (IQR: 0.05–0.22). For PTVs, the median JCI was 0.80 (IQR: 0.71–0.88) and the median GMI was 0.04 (IQR: 0.02–0.12). In one case, the iGTV GMI = 1 (implying tumour was completely missed), and in 3 other cases, GMI was >0.5 (implying at least 50% tumour miss). We failed to observe a close correlation between contoured volumes (iGTV, iPTV) and indices (Fig. 1). Indeed, the Spearman’s rho calculation results were as follows: iGTV jciGTV Spearman’s rho = −0.0714, iGTV gmiGTV Spearman’s rho = −0.3590, iPTV jciPTV Spearman’s rho = −0.1464 and iPTV gmiPTV Spearman’s rho = −0.4011.

### Relationship of geometric indices with the clinical outcome

**Fig. 2:**
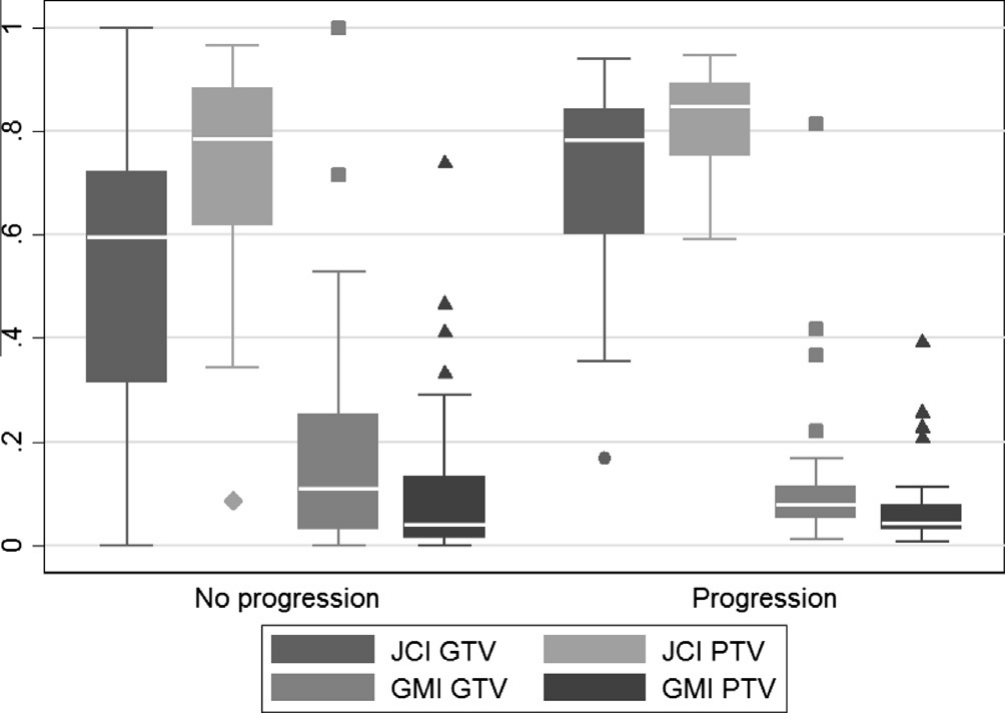
Distributions of the JCI and GMI indices by progression status at 9 months.

Of the 60 patients with high quality computer tomography imaging data, 58 had outcome data on PFS at 9 months. Fig. 2 shows the distributions of the GMI and JCI indices according to whether or not the patients progressed by 9 months. JCI GTV has the biggest difference in distribution between the two groups with higher JCI values in the group who progressed. ROC curves for GMIs and JCIs for PTVs and GTVs are shown in Supplementary Fig. 2. Only JCI PTV and GTV produced ROC curves with AUC > 0.6 and these had cut offs of 0.8 and 0.7 respectively providing the most correct classifications (60.34% and 70.69% respectively).

Table 1 shows the results of univariable and multivariable logistic regression for 9-month PFS. Of JCI GTV and PTV, JCI GTV showed the best univariable potential (*p* = 0.003) and was taken forward into the multivariable model where there continued to be strong evidence of its prognostic value. Patients with JCI GTV ⩾ 0.7 (implying at least 70% agreement between investigator and gold standard volume) had a 7.12 (95% CIs: 1.83–27.67, *p* = 0.005) higher odds of progressing by 9 months than those patients with JCI < 0.7.

### Review of PAF

Of the 74 planned patients, self-reported PAF was available for 70 and CD ROMs were submitted for 72, but the full DICOM data (CT slices, structures and dose) could only be processed and analysed in 66 cases. In general, there was good compliance to all aspects of dose prescription to PTV although deviations were observed (Table 2). Indeed, there were 7 minor deviations in the PTV receiving 95% of the prescribed dose falling below the required 99% with a minimum of 95.3%. PTV *D*_min_ was recorded on the PAF as between 90% and 93% for 10 patients (minor deviations) and <90% for 3 patients (major deviations, 4.5%). There was one minor deviation for the ICRU maximum defined dose of 107.9%. We failed to observe any deviation in the prescribed dose to the OARs.

**Fig. 3:**
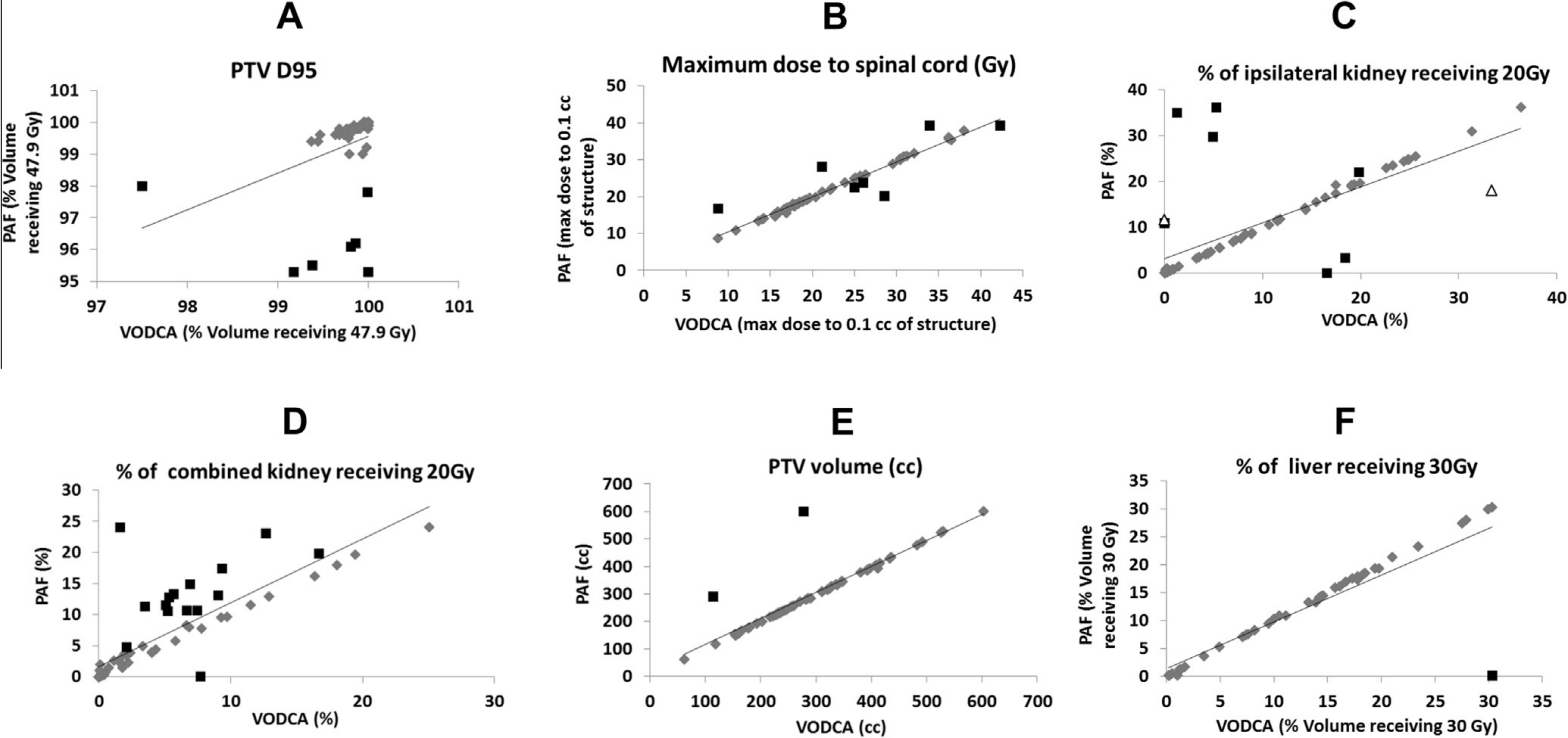
Illustration of the relationship between on-trial planning assessment form (PAF) value and the Visualization and Organization of Data for Cancer Analysis programme (VODCA) values in *n* = 66 cases, as indicated (A–F). Minor deviations are shown with black squares. Incorrect identification of the ipsilateral kidney (C) occurred in two cases (shown with circle).

Furthermore, we imported the planning values into VODCA and compared them to the PAF for the 66 cases to assess the accuracy of PAF completion and adherence to dose–volume constraints by the investigators (Fig. 3A–F). Seven minor deviations of dose constraints were identified from the PAF for PTV D95 (<99%), whereas one minor regarding the ICRU max (value 107.8). For the ipsilateral kidney receiving 20 Gy, seven minor deviations were found and incorrect identification of the ipsilateral kidney occurred in two cases (major deviations, 3%), which is significantly improved compared to the pre-trial case (20%) [7]. Other minor discrepancies were noted between PAF and VODCA regarding the maximum dose to the spinal cord (*n* = 7), combined kidney receiving 20 Gy (*n* = 15), PTV volume (*n* = 2) and liver receiving 30 Gy (*n* = 1).

## Discussion

In the present study we report the on-trial RTQA from the SCALOP trial. The median whole volume JCI for GTV was 0.64 (IQR: 0.43–0.82), and the median GMI was 0.11 (IQR: 0.05–0.22). For PTVs, the median JCI was 0.80 (IQR: 0.71–0.88) and the median GMI was 0.04 (IQR: 0.02–0.12). This appears to be consistent with the data shown in the recently reported pre-trial study [7]. In one case, the tumour was completely missed (GTV GMI = 1) and in 3 other cases, GMI was >0.5 (implying at least 50% tumour miss). No errors in GTV to PTV expansion were identified, and during treatment planning and delivery, incidence of major deviations were low.

The data recorded on the PAF were highly concordant with the actual planning data with no major discrepancies, suggesting that PAF could be used as a simple and effective aide- memoire to enhance protocol compliance.

We have recently reported the pre-trial RTQA of SCALOP where investigator contours of a test case were reviewed both quantitatively using geometric indices, and qualitatively (to subjectively evaluate acceptable/unacceptable over- and under-contouring) [7]. For contours that were considered qualitatively acceptable for the trial, the median whole tumour GMI and JCI were 0.3 (IQR 0.15–0.4) and 0.75 (IQR 0.7–0.8), respectively. The median geometric indices for GTV and PTV seen in on-trial patients were better than the test case, suggesting that overall, quality of tumour delineation was acceptable and that the pre-trial RTQA may have enhanced the quality of tumour delineation within the main trial. However, 4 cases had a tumour GMI in excess of 0.5, of which, the entire tumour was missed in one case – this suggests that although pre-trial RTQA can enhance the overall quality of tumour delineation, on-trial, real-time, central review is still necessary to ensure that the highest quality in tumour delineation is maintained.

Although retrospective, this is the first review of on-trial RTQA data from a clinical trial in LAPC and provides insight into the potential benefit of prospective review in this clinical setting. Experience from other setting/other tumour sites suggests that on-trial QA is essential to maintain integrity and quality of radiation trials. Protocol deviations including investigator error in post-operative tumour bed delineation was demonstrated in RTOG 9704, an adjuvant trial in pancreatic cancer, which also demonstrated a survival detriment in patients with major protocol violations [1]. In the recently reported NEOSCOPE study in oesophageal cancer, 86 patients were randomized to 2 pre-operative chemoradiation regimens. Eighty-three contours were reviewed, 39 (47%) of whom were reviewed prior to start of treatment and 44 (53%) were reviewed in a timely retrospective manner (prior to third fraction of RT). Nine cases (11%) required re-submission – inappropriate delineation of GTV and elective regional nodes were the most common cause of unacceptable deviations [12]. A recent study of brain metastasis has also demonstrated that real-time pre-treatment review of investigator contours and treatment planning limit unacceptable deviations [13]. Eighty-seven of 113 patients entered in the study underwent pre-treatment review. Twenty-one (24%) were noted to have unacceptable deviation, 18 of which were corrected on 2nd attempt and 2 on third attempt. Further 22 cases were reviewed retrospectively – 23% were found to have unacceptable deviations.

Prospective on-trial RTQA is a resource intensive, time-consuming and onerous process. On one hand – particularly in complex technical radiation trials (e.g. SBRT) or radiation with novel drug combination trials – there is risk of patient harm if appropriate radiotherapy is not undertaken; on the other hand, the RTQA process itself may unacceptably delay patient management. There are two potential options to achieve the best balance, either (a) to limit the trial to high volume centres to minimize the risk, or (b) undertake prospective review on a limited number of patients per centre, followed by timely retrospective or random reviews of subsequent cases. There are some data to support the former option from a non-pancreatic cancer clinical trial – the RTQA from one multi-centre Japanese study in oesophageal cancer (*n* = 142) shows that all unacceptable violations were from institutes which enrolled <7 patients [14].

Finally, we noted a strong relationship between JCI and survival, which remained as a highly significant independent prognostic marker on multivariate analysis even after adjusting for confounding variables, including tumour size. However, the optimum prognostic cut off of 0.7 that we found in this study would need to be validated in future studies. At this stage, we are unable to provide a suitable explanation why a high concordance between investigator and gold-standard contours predict for worse outcome – this observation that was counterintuitive to our expectations that lower JCI (ie a lower concordance) would predict worse survival. Of note, patients with higher JCI appeared to have significantly larger GTV compared to patients with lower JCI, albeit GTV lacked correlation with the 9-month PFS. A plausible hypothesis could be that better demarcated tumours (where there is likely to be a higher agreement amongst investigators, therefore higher JCI values) may reflect a more aggressive disease with worse prognosis. It is also possible that in patients who responded to the induction chemotherapy (and therefore had better prognosis), the tumour margins became less discernable leading to greater variation in contour delineation between the original investigator and review team. We are planning to pursue this issue in depth in the future through analysis of the correlation between CT image texture and clinical outcome.

The main limitation of this study was the unavailability of diagnostic images while performing central review, as diagnostic images were not routinely collected as part of this trial. It is possible that the discrepancies in outlines between the investigator and the review team may have been due to lack of adequate diagnostic information at the time of central review. However, the JCI and GMI values were similar or better than that seen at pre-trial test case, suggesting that this is unlikely to be the case.

In summary, the present study based on the SCALOP cohort highlights the need for on-trial prospective QA in future radiation trials in pancreatic cancer. PAF appears to be a simple tool to minimize protocol violation by providing an aide-memoire to protocol constraints and correlated well with the actual dose distribution. The relationship between JCI and survival shown needs to be explored further. Future trials are expected to benefit from the implementation of similar RTQA programmes that should include educational RT courses and real-time central review.

## Role of the funding source

The trial was developed on behalf of the NCRI Upper GI Clinical Studies Group and Cancer Research UK’s Clinical Trials Awards and Advisory Committee (CTAAC) approved the trial design and funded the study. CRUK had no role in study design, data collection, analysis or interpretation, or writing of this report.

## Conflicts of interest

We hereby confirm that there is no conflict of interest relevant to the present work.

## Figures and Tables

**Table 1:** Univariable and multivariable logistic regression of potential prognostic factors for disease progression by 9 months.

		Univariable analysis				Multivariable analysis			
		*n*	Odds ratio	95% CIs	*p*	*n*	Odds ratio	95% CIs	*p*
gsGTV	continuous	58	1.02	0.98–1.05	0.341	58	0.99	0.96–1.04	0.876
JCI GTV	<0.7	32	1.00			32	1.00		
⩾0.7	26	5.71	1.81–18.08	0.003	26	7.43	1.86–29.7	0.005
JCI PTV	<0.8	28	1.00						
⩾0.8	30	2.5	0.84–7.42	0.099				
Trial arm	Gem	35	1.00			27	1.00		
Cape	35	0.63	0.24–1.62	0.335	31	0.57	0.15-2.21	0.417
WHO PS	0	29	1.00			24	1.00		
1–2	41	1.41	0.54–3.73	0.484	34	1.45	0.39–5.43	0.583
Sex	Male	40	1.00			34	1.00		
Female	30	2.12	0.81–5.59	0.127	24	2.94	0.77–11.21	0.113
Age	<65	36	1.00			30	1.00		
⩾65	34	0.55	0.21–1.42	0.216	28	1.43	0.33–6.11	0.632
RT fractions	0–26	12	1.00			10	1.00		
27+	50	0.47	0.13–1.66	0.240	48	0.57	0.11–3.03	0.508

**Table 2:** Dose to PTV and OARs reported on the self-reported PAF.

Structure	Constraint	Mean	SD	Min	Max	Minor deviations	Major deviations
PTV vol (cc)	N/A	299.8	113.4	61.3	600.2	N/A	N/A
PTV D95 (%)	>99%	99.4	1.13	95.3	100	7	None
PTV *D*_min_ minimum (%)	N/A	93.9	1.70	86.7	96.4	10 below 93%	3 below 90%
ICRU max dose (%)	107%	103.8	1.90	100	107.9	1	None
Liver V30 (%)	<40%	12.6	8.2	0	30.3	None	None
Ipsilateral kidney V20 (%)	<40%	10.6	10.5	0	36.2	None	None
Combined kidney V20 (%)	<30%	6.5	6.8	0	24	None	None
Spinal cord PRV *D*_max_ (Gy)	<40 Gy	21.5	7.2	8.7	39.3	None	None

*Abbreviations:* N/A, not applicable; ICRU, International Commission on Radiation Units; SD, standard deviation.

## Footnotes

2 Digital Imaging and Communication in Medicine homepage: http://dicom.nema.org.
