# Supplementary material for: Comparison of investigator-delineated gross tumour volumes and quality assurance in pancreatic cancer: Analysis of the on-trial cases for the SCALOP trial
Source: Radiother Oncol. 2016 Aug;120(2):212–6. doi: 10.1016/j.radonc.2016.07.002 (PMC5013754; doi:10.1016/j.radonc.2016.07.002)
Supplement: Supplementary data [file mmc1.docx]

**SUPPLEMENTARY METHODS**

**RTTQA program**

The national NCRI Radiotherapy Clinical Trials Quality Assurance (RTTQA) team consisted of the chief investigator, a senior dosimetrist, a radiologist and RT-QA advisors (who were also clinical oncologists), working in conjunction with the Wales Cancer Trials Unit (WCTU) and the RTTQA group. The QA process for RT consisted of:

**(A) Pre-trial QA:**

1. Questionnaires completed by each centre: a) National QA Baseline questionnaire – detailed questions on available equipment, PTV Definition, Monitor Unit Check, Transfer of Plan to LINAC, DRRs, participant in-vivo dosimetry, and Shielding pre-treatment participant checks; b) National QA staff questionnaire – asked about experience of staff to be involved in RT on the trial. ; c) Trial specific questionnaire – to establish the extent of experience of RT for advanced pancreatic cancer and details of associated RT procedures across the centres. This was administered at the same time as the test cases are done.

2. Radiotherapy section of SCALOP protocol described the process for RT treatment outlining, planning and delivery for pancreatic cancer to aid the delivery of high quality RT. This was developed by a process including review by the SCALOP TMG, within the WCTU, peer-reviewed by UK Clinical Oncologists with a special interest in pancreatic cancer, peer-reviewed by an experienced Radiation Oncologist from the RTOG Trial group and within the NCRI RTTQA group.

3. A planning atlas was included in APPENDIX 8 of the protocol (http://www.wctu.org.uk/trial. php?trial=scalop).

4. One test case (DICOM CT data set along with a clinical summary) was sent to each participating clinician. They were required to provide the GTV, PTV, plan, and a Plan Assessment Form (PAF, supplementary figure 1) that assessed whether or not the plan conforms to the protocol. These were evaluated centrally by the SCALOP QA team and feedback was provided. Each clinical oncologist supervising radiotherapy within the SCALOP trial was required to complete the test case satisfactorily prior to entering participants in the trial. Each centre was required to submit one plan/PAF prior to entering participants in the trial.

**(B) On-trial QA:**

For every participant, clinicians at each centre were asked to complete a detailed PAF that was an integral part of the trials CRF. The main aim was to ensure concordance with the radiotherapy protocol, and allow real-time central review, providing an opportunity to identify major deviations prior to start of radiotherapy.

**Radiotherapy protocol**

The SCALOP trial protocol contained specific instructions on tumour delineation, treatment volumes, dose constraints and planning techniques that were to be followed for patients within the trial. All patients underwent contrast-enhanced planning computer tomography (CT) simulation with 200–300 mL water as oral contrast. The planning computer tomography (CT) scan was acquired in supine position following administration of 100ml of intravenous contrast (3ml/sec) in a Siemens Sensation Open CT scanner (Siemens, Erlangen, Germany) and 3mm slices were obtained using bolus tracking. The gross tumour volume (GTV) consisted of the primary tumour and any node with short axis diameter of 1 cm or more. The planning target volume (PTV) included the GTV with a margin of 2.0 cm in the craniocaudal direction and 1.5 cm in all other directions. Prophylactic irradiation of uninvolved (elective) regional nodes was not performed. A dose of 50.4Gy in 28 fractions was required to be prescribed to the International Committee on Radiation Units and Measurements (ICRU) 50 reference point, 1.8Gy per fraction, using at least 6MV photons. The exact number of beams, beam energy, beam arrangement and gantry angles were not explicitly defined but a single phase 3D conformal plan was required. The protocol stated that centres should aim to encompass the PTV with the 95% isodose, and that at least 99% of the PTV should receive 95% of the prescription dose (i.e. 47.9Gy). It was recommended that the minimum PTV dose should be >93% of the prescribed dose, but it was not considered to be a deviation if this was not achieved. The dose constraints were specified in the protocol (Supplementary Table 2). Intensity-modulated radiotherapy (IMRT) was allowed if previously developed and established as a departmental technique and the department had previously received credentials for IMRT by the NCRI RTTQA group. Centres were required to follow their local protocols as regards pre-treatment verification. As a minimum on-treatment verification should be carried out on the first 3 days of treatment and thereafter on a weekly basis. Key trial specific recommendations in the protocol included the use of both intravenous contrast and oral contrast/water during CT simulation, the need for advice from a gastrointestinal radiologist for GTV delineation and the compulsory completion and central review of the Plan assessment Form (PAF; Supplementary Figure 1) prior to initiation of RT on patients in the trial.

**Supplementary Figure 1.** The plan assessment form (PAF) is shown here.


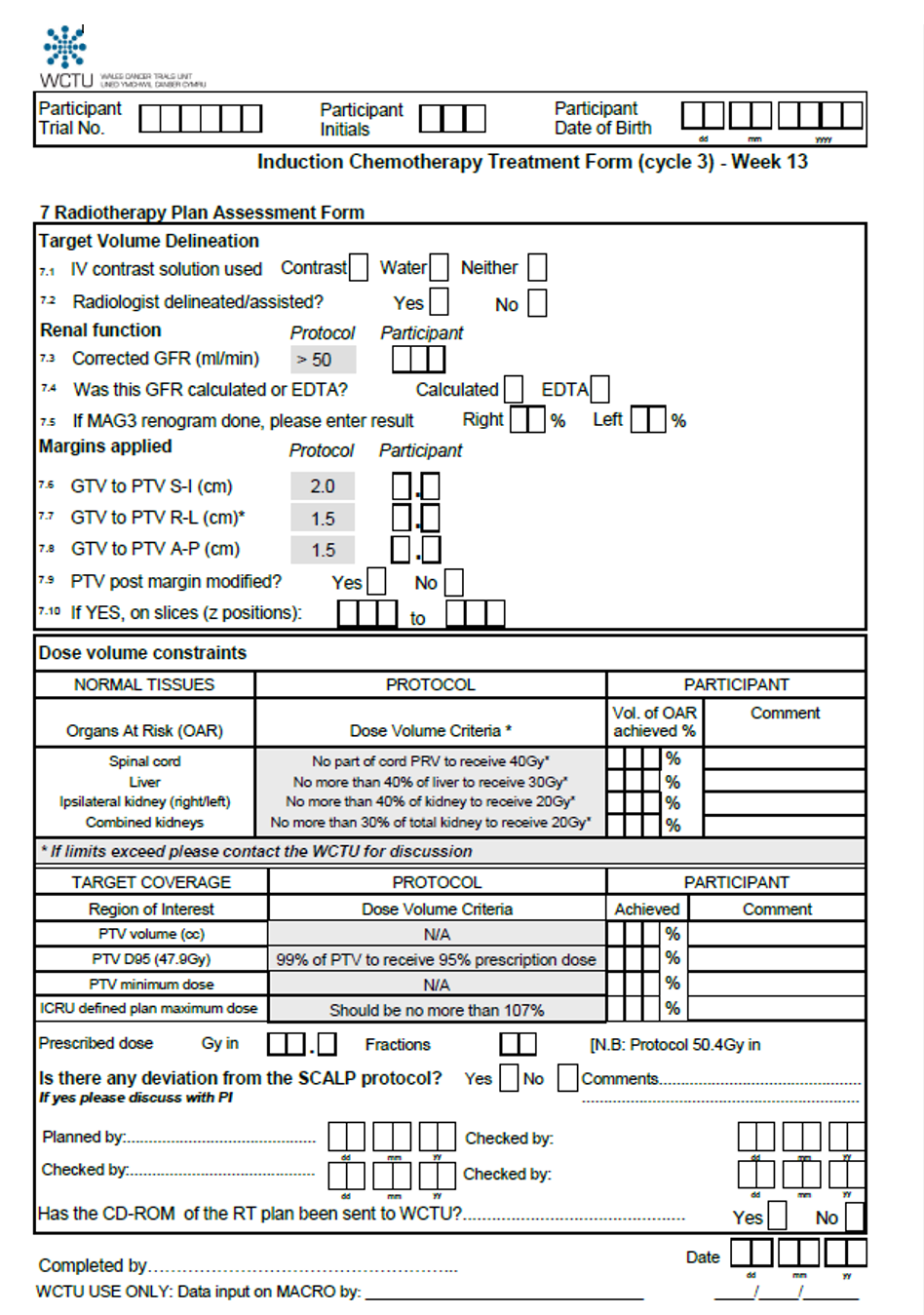


**Supplementary Figure 2.** ROC curves showing the sensitivity and specificity of different index and volume cut points for predicting disease progression by 9 months

| 1. **JCI GTV**   **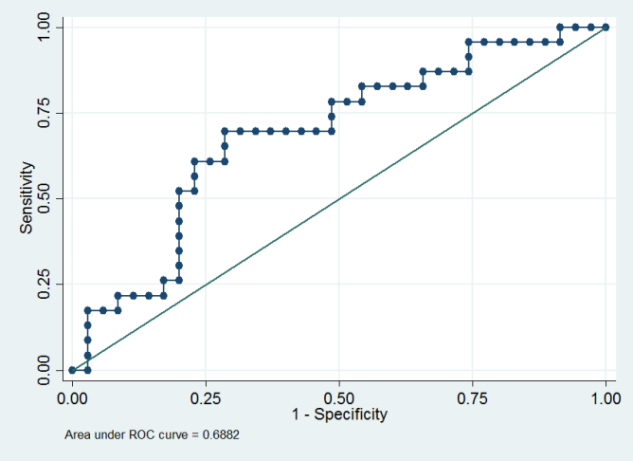** | 1. **JCI PTV**   **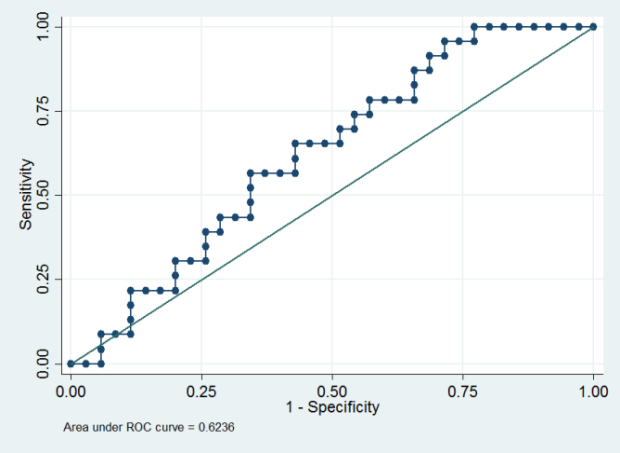** |
| --- | --- |
| 1. **GMI GTV**   **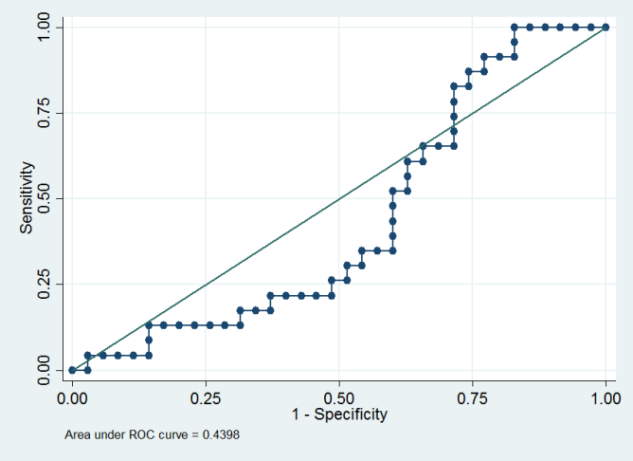** | 1. **GMI PTV**   **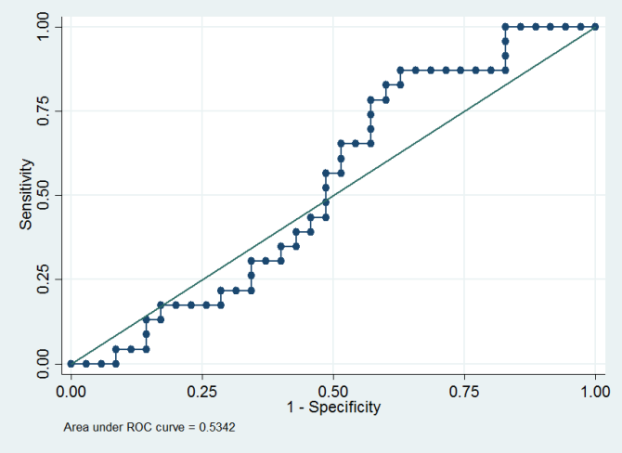** |
| 1. **Gold standard GTV**   **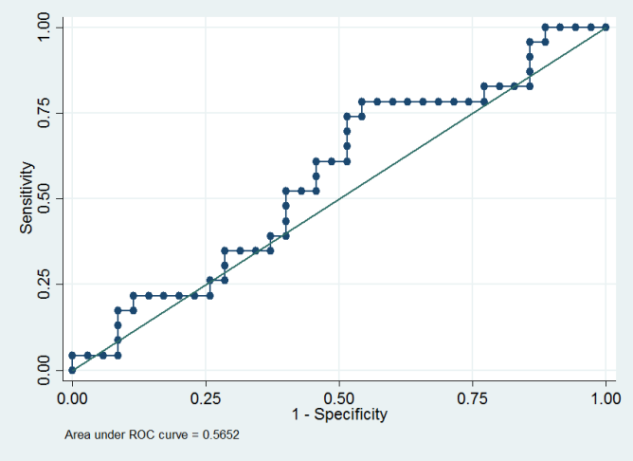** | 1. **Investigator GTV**   **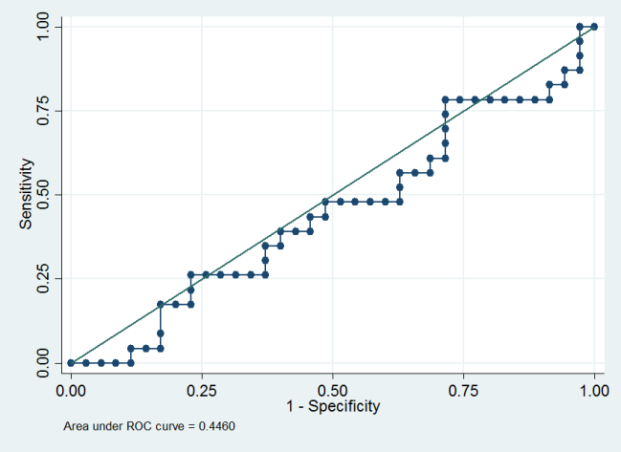** |

**Supplementary Table 1.** Formulas and descriptions of different metrics used for contour comparisons.

| **Conformity Index** | **Equation / Description** | |
| --- | --- | --- |
| ***Jaccard Conformity Index***  ***JCI***  *Range: 0 – 1; Ideal: 1* | $\frac{A\cap B}{A\cup B}$ | Amount of the gold standard contour covered by the investigating contour as a fraction of their encompassing volumes. |
| ***Geographical Miss Index***  ***GMI***  *Range: 0 – 1; Ideal: 0* | $\frac{B-\left(A\cap B\right)}{B}$ | Amount of the gold standard contour missed by the investigating contour as a fraction of the gold standard contour. |
| *Abbreviations:* A = investigator contour; B = gold standard contour;  A$\cap$B = intersection of A and B; A$\cup$B = union of A and B. | | |

**Supplementary Table 2.** Dose-Volume constraints and trial deviations for the SCALOP trial

| **Region of interest / organ at risk** | **Dose Constraint** | **Further detail** | **Minor variation** | **Major Deviation (acceptable)** | **Major Deviation (unacceptable)** |
| --- | --- | --- | --- | --- | --- |
| PTV | V95% (47.9Gy)> 99.0% | More than 99% of the PTV volume to receive 95% of the prescribed dose | ≥95% | ≥90% | < 90% |
| PTV Dmin | N/A | Recommended to be >93% | <93% | <90% |  |
| PTV Dmin | ≤107% | Region considered clinically meaningful if minimum diameter exceeds 15mm | ≤110% | ≤113% | >113% |
| Spinal Cord planning risk volume | V40 Gy <0% | Maximum dose to any part of the spinal cord PRV is 40Gy | V42Gy <0% | V45Gy <0% | Any cord receiving >45Gy |
| Liver | V30 Gy < 40% | No more than 40% of the liver to receive 30Gy | V30Gy ≤ 45% | V30Gy ≤ 50% | V30 > 50% |
| Ipsilateral Kidney (or for central tumours, kidney receiving the higher dose) | V20 Gy < 40% | No more than 40% of the kidney receiving the highest dose to receive 20Gy | V20Gy ≤ 45% | V20Gy ≤ 50% | V20 > 50% |
| Combined Kidneys | V20Gy < 30% | No more than 30% of the combined kidney to receive 20Gy. | V20Gy ≤ 35% | V20Gy ≤ 40% | V20 > 40% |
